# Correlating Microbial Dynamics with Key Metabolomic Profiles in Three Submerged Culture-Produced Vinegars

**DOI:** 10.3390/foods14010056

**Published:** 2024-12-28

**Authors:** Juan J. Román-Camacho, Inés M. Santos-Dueñas, Isidoro García-García, Teresa García-Martínez, Rafael A. Peinado, Juan C. Mauricio

**Affiliations:** 1 Department of Agricultural Chemistry, Edaphology, and Microbiology, Microbiology Area, Agrifood Campus of International Excellence ceiA3, University of Córdoba, 14014 Córdoba, Spain; b32rocaj@uco.es (J.J.R.-C.); mi2gamam@uco.es (T.G.-M.); mi1gamaj@uco.es (J.C.M.); 2 Department of Inorganic Chemistry and Chemical Engineering, Chemical Engineering Area, Agrifood Campus of International Excellence ceiA3, Nano Chemistry Institute (IUNAN), University of Córdoba, 14014 Córdoba, Spain; ines.santos@uco.es; 3 Department of Agricultural Chemistry, Edaphology, and Microbiology, Agricultural Chemistry Area, Agrifood Campus of International Excellence ceiA3, University of Córdoba, 14014 Córdoba, Spain; rafael.peinado@uco.es

**Keywords:** *Acetobacter*, *Komagataeibacter*, metabolomics, Nitrososphaeraceae, raw material, vinegar

## Abstract

Although vinegar is a product obtained by a well-known bioprocess from a technical point of view, the complex microbiota responsible for its production and their involvement in the organoleptic profiles are not clear yet. In this work, three acetification profiles in submerged culture using both synthetic and raw materials from Andalusia (Spain) were characterized by metagenomic (16S rRNA amplicon sequencing) and metabolomic tools (stir-bar sorptive extraction with thermo-desorption coupled to gas chromatography–mass spectrometry (SBSE-TD-GC−MS) and high-performance liquid chromatography (HPLC)). A total of 29 phyla, 208 families, and many more genera were identified, comprising bacteria and archaea as well as 75 metabolites, including minor volatile compounds, amino acids, biogenic amines, and other nitrogenous compounds. It can be concluded that *Komagataeibacter* and *Acetobacter* were not only the predominant genera but also the ones that most influenced vinegar metabolite profiles by using different metabolic strategies for mutual collaboration, and together with other microbial groups, some of them were previously practically unknown in vinegar. These results can be of interest not only to deepen the basic knowledge about vinegar but also to the vinegar industry by elucidating microbial succession and the key associated metabolites.

## 1. Introduction

Vinegar, a fermented foodstuff discovered over 5000 years ago, is used worldwide as an important food preservative, seasoning, and flavoring additive [1,2]. Beyond its culinary uses, vinegar also holds a significant value in health care, prevention, and disease treatments due, in great part, to its chemical and nutritional composition, which includes main components like acetic acid, exerting a healthy influence on consumers [3].

Vinegar production includes a biotransformation step in which the oxidative activity of acetic acid bacteria (AAB) allows the conversion of the ethanol of the medium into acetic acid [4,5]. However, it is currently known that the microbial composition of vinegar consists of complex cultures in which different taxonomic groups may cohabitate, mainly members of the family Acetobacteraceae [6,7,8]. *Acetobacter*, *Gluconacetobacter*, and *Komagataeibacter* species are frequently found during the elaboration of European vinegar because of their particular growth conditions and efficient oxidative ability [5,7].

On the other hand, the raw material used for vinegar elaboration highly depends on the producing region; while in Asia, the acetification substrates usually consist of liquor obtained from cereals or grains (rice, wheat, millet, and sorghum), in Europe, those from fruits (grapes, apples, and berries, among others) and spirits predominate [9,10]. Certainly, in the Mediterranean region, the particular climate and soil factors allow the growing of native raw materials that are the basis for obtaining high-quality acetification substrates such as musts, wines, and beers [11,12]. Regarding production systems, submerged culture is usually imposed on solid-state fermentation and surface culture in Europe to obtain a higher acetification yield and process rate [4,13,14]. Submerged culture implies the use of bioreactors that continuously supply very fine air bubbles into the medium as a high-efficiency aeration mechanism [2,4]. Here, the semi-continuous working mode usually prevails as one of the most common; in this mode, the process runs in cycles, each concluding when the ethanol concentration in the medium decreases up to a certain value. At that time, the reactor is partially unloaded until it reaches a certain volume; the remaining content is used as inoculum for the next cycle, which starts by introducing a fresh medium. Semi-continuous mode is preferred over others (continuous and batch modes) because it better supports the growth of mixed cultures that are auto-adapted to both substrate and product [14,15].

Recent studies have focused on the correlation of the existing microbiota with metabolomic and sensory profiles of some types of vinegar in Asia, such as Hainan dregs vinegar [16], Shanxi aged vinegar [17,18], and jujube vinegar [6], as well as other fermented foods, including xiaoqu baijiu and minced pepper, among others [19,20,21]. However, studies on European vinegars made from typical raw materials have largely ignored these aspects. Therefore, it is necessary to establish the basis for a better understanding of the diversity and role of the microbial communities present, whose activity may release metabolites into the medium, influencing the organoleptic properties of these appreciated products.

To address these issues, the implementation of multi-omic strategies is enabling the massive analysis of macromolecules and metabolites of interest for the study of fermented foods contributing multiple aspects such as the composition, aroma, flavor, and bio-healthy properties, which, applied to the vinegar industry, may have a direct impact on the improvement of production procedures, quality, and consumers’ acceptance [22,23]. Diverse authors have used metagenomic and metabolomic approaches for the study of mostly traditional vinegar making, such as amplicon and shotgun sequencing [11,13,24] along with gas chromatography−mass spectrometry (GC−MS) [17,25,26], high-performance liquid chromatography (HPLC) [27], capillary electrophoresis−mass spectrometry (CE−MS) [28], and nuclear magnetic resonance spectrometry (NMR) [9] for metabolomics. However, the depth currently achieved in these studies and, above all, in Europe-produced vinegar, particularly regarding the evolution of microbial diversity and its correlation with metabolite profiles, is still insufficient.

In this study, three alcoholic substrates were selected for vinegar making by submerged culture and semi-continuous operating mode with a synthetic alcohol-based medium and two raw materials from Andalusia (Spain): a dry fine wine and a craft dark beer. The aim was to characterize and compare the evolution of acetification profiles not only from a technical point of view but also from microbial succession and its correlation with the metabolome. 16S rRNA amplicon sequencing, as well as stir-bar sorptive extraction with thermo-desorption and GC−MS (SBSE-TD-GC–MS) and HPLC, were implemented. To our knowledge, there is no background on research applying a multi-omic strategy (metagenomics─metabolomics) of vinegar obtained from raw materials and industrial production systems typical of Europe and with the level of detail intended here. The findings obtained may provide valuable insights applicable to the vinegar industry.

## 2. Materials and Methods

### 2.1. Raw Materials

Three raw materials of diverse alcoholic origins were used as acetification media. The first substrate consisted of a synthetic alcohol-based medium (AM) prepared in the laboratory according to Llaguno’s recipe [29], supplemented with peptone (0.5 g/L) and yeast extract (0.25 g/L). The other two substrates were traditionally elaborated: a dry fine wine (FW) from the Montilla-Moriles region (Bodegas Alvear S.A., Montilla, Córdoba, Spain) and a dark craft beer (CB), highly sugared (Mahou-San Miguel, Córdoba, Spain). In all cases, the ethanol content of the media was adjusted to the working conditions, around 10% *v*/*v*. The initial acidity level (expressed as a mass percentage of acetic acid relative to the volume of the medium) was 0.1 ± 0.1% *w*/*v* for AM and 0.2 ± 0.1% *w*/*v* for FW and CB. Additional information about the chemical and nutritional composition of the raw materials is available in Román-Camacho et al. [30].

### 2.2. Starter Inoculum

A sample of the culture medium collected from the final stage of an industrial bioreactor producing wine vinegar (UniCo Vinagres y Salsas, S.L.L., Doña Mencía, Córdoba, Spain) was used as the original microorganism source. The inoculum sample was adapted with synthetic alcohol-based medium (AM) for its use as the starter culture for the three acetification profiles. Some preliminary cycles were necessary to calibrate the probes and adapt the microorganisms to each medium.

### 2.3. Fermentation Conditions

**Figure 1:**
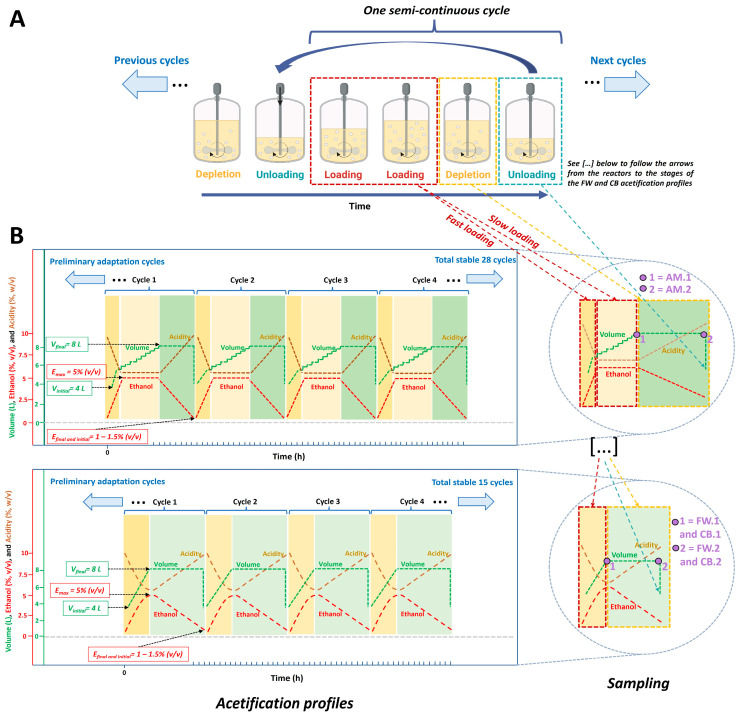
(**A**) Diagram describing the different phases of semi-continuous cycles in a submerged acetification process. (**B**) Scheme of the acetification profiles of the different substrates used, including sampling times. AM, synthetic alcohol-based medium profile; AM.1, sampling at the end of the loading phase; AM.2, sampling just before the unloading phase (**top panel**). FW, fine wine profile; CB, craft beer profile; FW.1 and CB.1, sampling at the end of the loading phase; FW.2 and CB.2, sampling just before the unloading phase (**bottom panel**).

Acetification profiles were performed using a fully automated 8 L Frings reactor (Heinrich Frings GmbH & Co., KG, Bonn, Germany). The bioreactor operated in a semi-continuous mode in which the ethanol in the medium was gradually reduced to a specified concentration (1.0% *v*/*v*); when this ethanol concentration was reached, approximately 50% of the reactor content was unloaded (4 L). Afterward, the bioreactor was refilled until reaching the working volume of 8 L, while ensuring that a preset ethanol concentration of 5% *v*/*v* was not overstepped. For this purpose, the loading necessary to reach the working volume (8 L) may present two phases: the first is a fast loading phase in which a volume of fresh medium is added until ethanol concentration (5% *v*/*v*) is reached, and from this moment on, a slow loading stage is started in which the necessary volume is discontinuously added so as not to exceed the aforementioned ethanol content. This occurred for the acetification profile of synthetic-based alcohol medium; this working system is shown in Figure 1. A constant temperature of 31 °C, a loading rate of 1.3 ± 0.1 L/h, and an airflow rate of 7.5 L/(h L medium) were applied in all profiles.

### 2.4. Sampling

Before starting acetification, samples for metagenomic analysis were taken from the working starter culture (inoculum); additionally, samples for metabolomic analysis were also taken directly from the raw materials without inoculation (AM.0, FW.0, and CB.0). After inoculation and before sampling for full analysis, a period of several preliminary cycles to achieve stable and repetitive semi-continuous acetification profiles was conducted. Once repetitive cycles were achieved for each profile, numerous stable cycles were developed for sampling: 28 stable cycles for the acetification profile of synthetic alcohol-based medium and 15 for the acetification profiles of fine wine and craft beer, respectively. Samples of the acetification profiles were then taken first at the end of the loading phase, reaching the reactor working volume (AM.1, FW.1, and CB.1), and then at the end of the fermentation phase, just before unloading, when the acidity level was the highest throughout the cycle (AM.2, FW.2, and CB.2) (see Figure 1B). Three or four biological replicates (samples) were harvested over different stable cycles of each acetification profile.

### 2.5. Analytical Methods

System variables including the fermenting volume, ethanol concentration, and temperature were continuously monitored through an EJA 110 differential pressure probe (Yokogawa Electric Corporation, Tokyo, Japan) and an Alkosens R probe (Heinrich Frings GmbH & Co., KG, Bonn, Germany), and the temperature reading was obtained from the same aforementioned ethanol probe. The automated system enabled uninterrupted data recording and validated the method’s high reproducibility. Total acidity (expressed as % *w*/*v,* g of acetic acid/100 mL of medium) was measured using acid-base titration with 0.5 N NaOH. The total and viable cell concentrations were directly quantified with an optical microscope (BX51 model, Olympus, Hamburg, Germany) and a Neubauer chamber (Brand^TM^, 7178-10 model, Brand, Wertheim, Germany) with a depth of 0.02 mm and a rhodium-coated bottom [31]. These measurements were taken exclusively at sampling moments.

### 2.6. Metagenomics

#### 2.6.1. Samples Processing

A volume of 300 mL, subsequently divided into six fractions of 50 mL each, was collected per vinegar sample at times indicated in Section 2.4. Cell samples were obtained after double washing with cold, sterile, distilled water and centrifugating, and they were later stored at −80 ° C. Genomic DNA (gDNA) from each cell sample was extracted by using a quick genomic bacterial DNA extraction kit (Bio Knowledge Lab, S.L., Córdoba, Spain) following the instructions provided by the manufacturer. gDNA was purified and quantified by NanoDrop ND 1000 spectrophotometer (Thermo Fisher Scientific, Boston, MA, USA).

#### 2.6.2. 16S rRNA Sequencing

The V3-V4 region from 16S rRNA genes was amplified by the specific set of primers 341F (5’-CCTACGGGNGGCWGCAG-3’)-806R (5’- GGACTACHVGGGTWTCTAAT -3’) with barcodes [32,33]. PCR reactions and library construction were carried out as described in Román-Camacho et al. [11]. The amplicon was sequenced on the Illumina paired-end platform to generate 250 bp paired-end raw reads. The resulting raw reads were processed by using QIIME2 v2020.8 (https://qiime2.org/) [34]. Reads (fastq) obtained after Illumina amplicon sequencing were denoised using the DADA2 package “https://github.com/benjjneb/dada2 (accessed on 7 February 2021)” by conducting three steps: (1) trimming and truncating low-quality regions, (2) dereplicating reads, and (3) chimera filtering [35]. After denoising, forward and reverse reads were merged into a fasta sequence, dereplicated, and assigned to an ID, considering them as amplicon sequence variants (ASVs). The list of reads obtained is available in Table S1 (see Supplementary Materials). ASVs were grouped in operational taxonomic units (OTUs) through the de novo clustering method from vsearch [36]. Clustering was carried out at 97% identity to generate 97% OTUs.

### 2.7. Metabolomics

#### 2.7.1. Minor Volatile Compounds Analysis

A volume of 10 mL, in triplicate, was collected per sample in Falcon tubes and stored at −20 °C for minor volatile compounds determination (<10 mg/L). The analysis was conducted in a two-step process by applying stir-bar sorptive extraction, followed by thermo-desorption and gas chromatography–mass spectrometry (SBSE-TD-GC–MS) as described by Martínez-García et al. [26]. For the extraction, a volume of 1 mL of each vinegar sample was 1:10 diluted with distilled water in a 10 mL vial and 0.1 mL of ethyl nonanoate (0.45 mg/L) as internal standard. Samples were then stirred by using a polydimethylsiloxane (PDMS)-coated stir bar (Twister) with 0.5 mm film thickness and 10 mm length (Gerstel GmbH, Mülheim an der Ruhr, Germany) at 1500 rpm for 100 min at 20 °C. The Twister was removed and transferred into a desorption tube for chromatographic analysis.

The second step entailed the determination of the volatile compounds in a GC–MS equipped with a Gerstel TDS 2 thermo-desorption system. Desorption tubes were heated at 280 °C for 10 min to release volatile compounds attached to the Twister and retained in a TENAX adsorption tube. The desorption was conducted at 25 °C in a CIS 4 PTV cooling system, containing the TENAX adsorption tube, with a subsequent temperature increase up to 280 °C to release volatiles. GC–MS, equipped with an Agilent-19091S capillary column (30 m × 0.25 mm i.d. and 0.25 μm film thickness), was operated at 50 °C for 2 min and 190 °C for 10 min. Helium was used as carrier gas at a flow rate of 1 mL/min. The mass detector worked in scan mode at 1850 V and checked the mass from 39 to 300 m/z.

Volatile compound identification was performed by using retention times of standards injected under the same chromatographic conditions as samples and the Wiley N7 spectral library. Quantification was performed using calibration curves of the standard.

#### 2.7.2. Nitrogenous Compounds Analysis (HPLC)

The quantification of amino acids, biogenic amines, and ammonium ions was carried out using a modified derivatization technique involving diethyl ethoxymethylenemalonate (DEEMM), as outlined by Gómez-Alonso et al. [37]. A volume of 5 mL, in triplicate, was collected per sample in Falcon tubes and stored at −20 °C. The derivatization of compounds was achieved by combining 0.250 mL of untreated sample, 0.250 mL of Milli-Q water, 0.500 mL of methanol, 0.750 mL of 1 M borate buffer (pH = 9), 0.050 mL of L-2-aminoadipic acid (1 g/L) as an internal standard, and 0.003 mL of DEEMM in a tube. This mixture was then subjected to ultrasonication for 30 min and heated at 70 °C for 2 h.

The HPLC analysis was conducted using an HPLC 1260 Infinity model (Agilent, Santa Clara, CA, USA) equipped with a 25 cm ACE C18-HL column with a particle size of 5 μm (250 mm × 4.6 mm), which was maintained at 16 °C. The separation was achieved using a binary gradient of phases A and B [37]. For detection, a photodiode array detector was utilized with monitoring at 280 nm.

### 2.8. Statistical Treatment

IBM SPSS Statistics (version 28.0) software was applied to conduct statistical analyses including the detection of significant differences by analysis of variance (ANOVA), principal coordinates analysis (PCoA) by applying the statistic Bray–Curtis distance, bivariate correlations with confidence intervals (Spearman coefficients), hierarchical clustering (by Ward’s method), and principal component analysis (PCA).

## 3. Results

### 3.1. Description of System Variables

The mean values and standard deviations of the system variables for monitoring the three acetification profiles are shown in Table 1. As can be seen, the AM profile was slower (AM.2, 28.9 ± 2.6 h) than the rest, and that of FW was the fastest (FW.2, 21.4 ± 0.1 h). It is worth noting that the CB profile was adjusted to a lower working volume (CB, 7.0 ± 0.2 L) because of the excessive foaming. Regarding ethanol concentration, the AM profile exhibited a lower ethanol disappearance rate than the rest, as in this case, the pre-set ethanol concentration (AM.1, 5.0 ± 0.1% *v*/*v*) was reached before the working volume (AM.1, 8.0 ± 0.1 L); therefore, an additional discontinuous loading phase was necessary. Regarding acidity, the FW profile showed the highest increase during the fermentation phase, from 4.3 ± 0.0 to 7.9 ± 0.2% *w*/*v*, and the CB profile, coming from a craft beer with high sugar content, provided the lowest increase, from 4.2 ± 0.0 to 6.8 ± 0.7% *w*/*v*. Moreover, it is interesting to note that the highest content of viable cells was found throughout the AM profile (Table 1). The acetification efficiency, represented by the variables *r_A_* and *p_A_*, indicated that the FW and CB profiles were more efficient in terms of production than the AM profile, especially the FW profile (*r_A_*_,_ 1.9 ± 0.0 g acetic acid/(L h); *p_A_*, 15.2 ± 0.5 g acetic acid/h).

### 3.2. Microbial Diversity Throughout Acetification Profiles

The number of high-quality unique ASVs obtained after carrying out strict quality controls was 12,443, of which 5998 unique OTUs were detected at 97% identity in, at least, one out of the total samples analyzed by amplicon metagenomics (n = 20 samples). A more detailed description of the total identified OTUs can be found in Table S2 (see Supplementary Materials).

**Figure 2:**
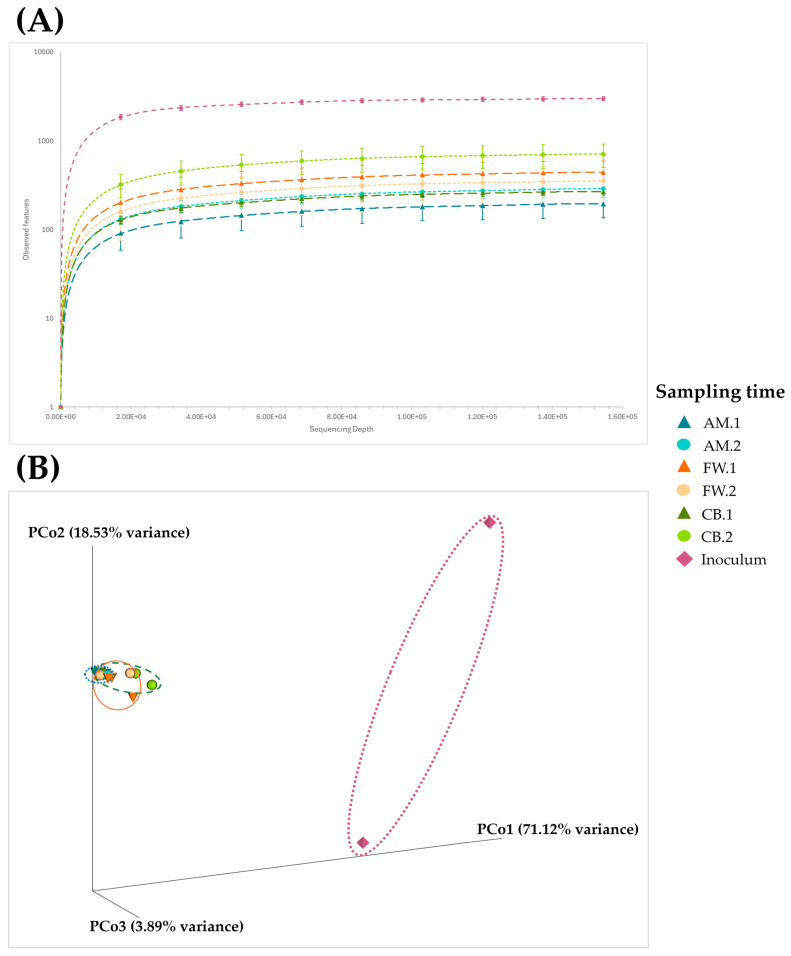
(**A**) Alpha rarefaction plot represented by the observed features as the alpha biodiversity quantitative index. Dots show mean values and their standard deviation of observed features metric, representing the distribution for each sample group at each even sampling depth. The line chart connects the dots of the metric distribution across sampling depths. (**B**) Principal coordinates analysis (PCoA) by applying the statistic Bray–Curtis distance as the quantitative measure of community dissimilarity for beta biodiversity analysis. Samples are represented with different shapes and colors in a tri-dimensional matrix, and the distance between them indicates diversity between samples (more distance, higher diversity). Synthetic alcohol-based medium (AM); craft beer (CB); fine wine (FW); end of loading phase (AM.1, CB.1, and FW.1); just before unloading phase (AM.2, CB.2, and FW.2).

Based on the number of OTUs detected in the samples and their distribution, a comparative biodiversity analysis was performed. Alpha diversity, i.e., biodiversity within samples, was determined by applying three quantification diversity indexes, including observed features, Shannon, and Simpson indexes (see Table 2). For data visualization, the observed features index was selected to represent the biodiversity of samples by an alpha rarefaction plot (see Figure 2A). Two distinct groups can be observed: the inoculum samples, which were much more diverse, and the acetification profile samples. Regarding the latter samples, differences were also observed in all indexes. The AM profile exhibited a lower overall diversity than the rest of the profiles (FW and CB), although it increased after the fermentation phase (AM.2). For the CB profile, diversity was also considerably higher at the final moments before unloading (CB.2), while the diversity of the FW profile was more stable and slightly higher at the end of loading stage (FW.1) than just before unloading (FW.2).

Beta diversity was analyzed by the statistic Bray–Curtis distance as a quantitative measure of community dissimilarity and represented by a principal coordinates analysis (PCoA) (see Figure 2B). A total of five principal coordinates (PCo) were obtained, of which the three explaining up to 93.54% of the variance were represented in a three-dimensional matrix. In this case, and similar to the alpha diversity analysis, significant differences can be appreciated between the inoculum samples and those of the acetification profiles due to the wide distance observed in the matrix. Samples of the different stages of the AM profile (AM.1 and AM.2) seemed to show very small differences in biodiversity due to their proximity in the matrix, in which it was difficult to even differentiate samples from each other. The distribution of samples of the FW profile (FW.1 and FW.2), although close to each other, did not seem to be highly determined by the evolution of the acetification process, while in the CB profile, samples were grouped slightly more concerning the sampling times (CB.1 and CB.2). Because the Bray–Curtis distance is a quantitative measure, the results may indicate that the OTUs’ frequency plays a crucial role in microbial diversity. Moreover, the evolution of the acetification process, considering that the starting point was a significantly different inoculum, seemed to have more influence than the use of different raw materials for vinegar making regarding microbial diversity.

### 3.3. Microbial Dynamics Throughout Acetification Profiles

**Figure 3:**
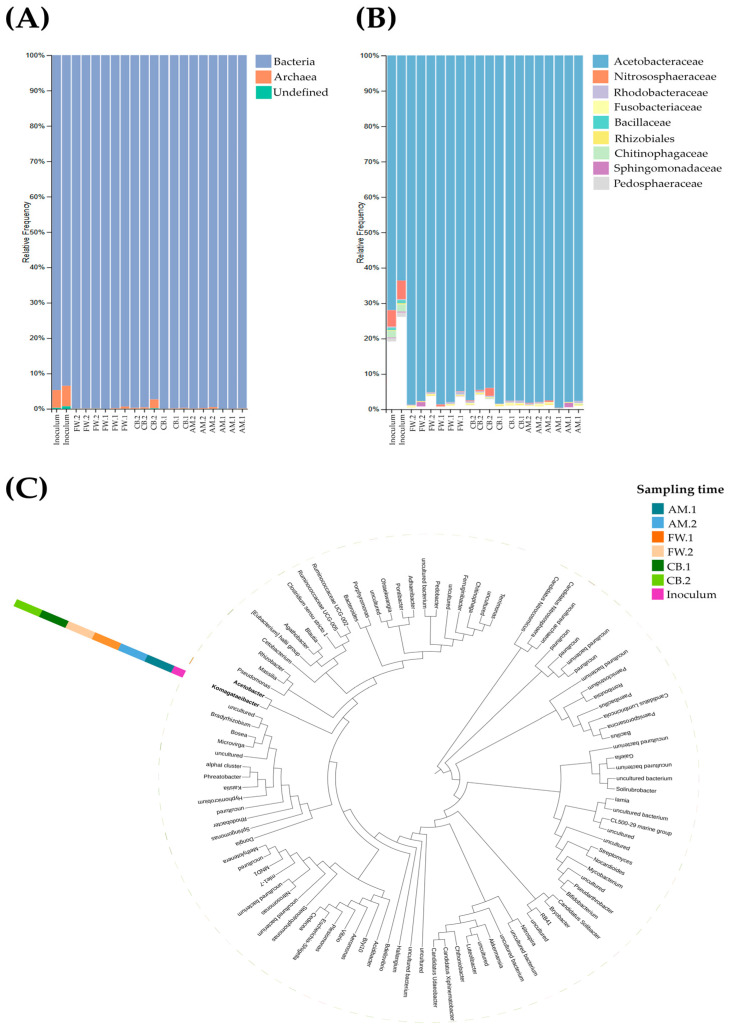
(**A**,**B**) Taxonomy bar plot representing the relative frequency (%) of the total OTUs associated with each taxon at the domain and family level, respectively. The set of taxa assigned to each of the total OTUs, including the main taxa and those conforming to a minor fraction (see uncolored section in (**B**)), can be consulted in Table S2 (see Supplementary Materials). (**C**) Phylogenetic tree collapsed at the genus level with frequency at each sampling time. Synthetic alcohol-based medium (AM); craft beer (CB); fine wine (FW); end of loading phase (AM.1, CB.1, and FW.1); just before unloading phase (AM.2, CB.2, and FW.2).

Assigning taxa to OTUs identified in vinegar samples allowed the taxonomic composition of the metagenome to be determined and thus also the microbial dynamics along the three profiles. Figure 3 shows the microbial succession throughout each acetification profile, allowing comparison between them at three taxonomic levels. Bar plots show the relative frequency (%) of main taxonomic groups contributing to each sample at the domain and family level. At the domain level (see Figure 3A), the starting inoculum was composed of an average of 94.08% bacteria and 5.38% archaea, while 0.54% were determined as unassigned OTUs. During the course of acetification profiles, the frequency of bacteria increased to limits above 99% in most cases, regardless of the raw material or the phase. As a consequence, the frequency of archaea decreased considerably; in the AM profile, the mean frequency of archaea increased from 0.07% in AM.1 to 0.25% in AM.2, while the FW profile decreased from 0.26% in FW.1 to 0.10% in FW.2. Interestingly, the increase throughout the fermentation of the CB profile was significantly greater, going from 0.13% in CB.1 to 1.05% in CB.2. The frequency of unassigned OTUS was practically insignificant. At the family level (see Figure 3B), Acetobacteraceae, including acetic acid bacteria (AAB), was predominant, representing 67.78% of the inoculum microbial community, which subsequently increased to frequencies between 95 and 98% in the three profiles. The family that mainly represented the archaea group was Nitrososphaeraceae, contributing to 4.97% of the starting inoculum, which subsequently dropped to frequencies between 0.1 and 0.2% at sampling times except for CB.2, where it contributed almost 1% (0.96%) out of the total. At lower frequencies, the following more representative families of the microbial community were Rhodobacteraceae, Fusobacteriaceae, Bacillaceae, Rhizobiales (order, family undetected), Chitinophagaceae, Sphingomonadaceae, and Pedosphaeraceae. In total, these nine taxonomic groups covered frequencies of 70–80% in starting inoculum and >97% in acetification profiles. Nevertheless, it is worth noting that the total number of taxa detected by 16S rRNA gene amplicon sequencing was 29 phyla and 208 families, comprising bacteria and archaea. The full list of identified OTUs and their corresponding assigned taxon, including those comprising the minor fraction of taxa (see uncolored section in Figure 3B), is available in Table S2 (see Supplementary Materials).

Upon deeper analysis of microbial succession, a phylogenetic tree that was collapsed at the genus level was constructed for the top 100 OTUs at each sampling time (see Figure 3C). The dominant genus, far above the rest, was *Komagataeibacter*, which was consistent at all acetification sampling times regardless of the raw material and cycle time; interestingly, the *Komagataeibacter* frequency was lower in the starting inoculum. The second most abundant genus was *Acetobacter*, which is closely related to *Komagataeibacter*, as both are AAB belonging to the abundant Acetobacteraceae family (Figure 3B). *Acetobacter* was mainly found in FW profile samples. At lower frequencies, OTUs belonging to numerous bacterial genera were identified, such as *Pseudomonas*, *Rhizobacter*, *Cetobacterium*, *Bacteroides*, *Pedobacter*, *Bacillus*, *Streptomyces*, *Bifidobacterium*, *Nitrospira*, *Acidibacter*, *Aeromonas*, *Vibrio*, *Nitrosomonas*, *Sphingomonas*, and *Rhodobacter*, among others, forming the phylogenetic tree. One clade (see top right side) proposed “*Candidatus Nitrososphaera*” and “*Candidatus Nitrosocosmicus*” as archaea representatives besides a third undefined group (uncultured archaeon). Other members of the phylogenetic tree were also defined as “uncultured” because 16S rRNA sequencing could not identify them down to the genus level.

### 3.4. Comparison of Metabolite Profiling in Vinegar Making

**Figure 4:**
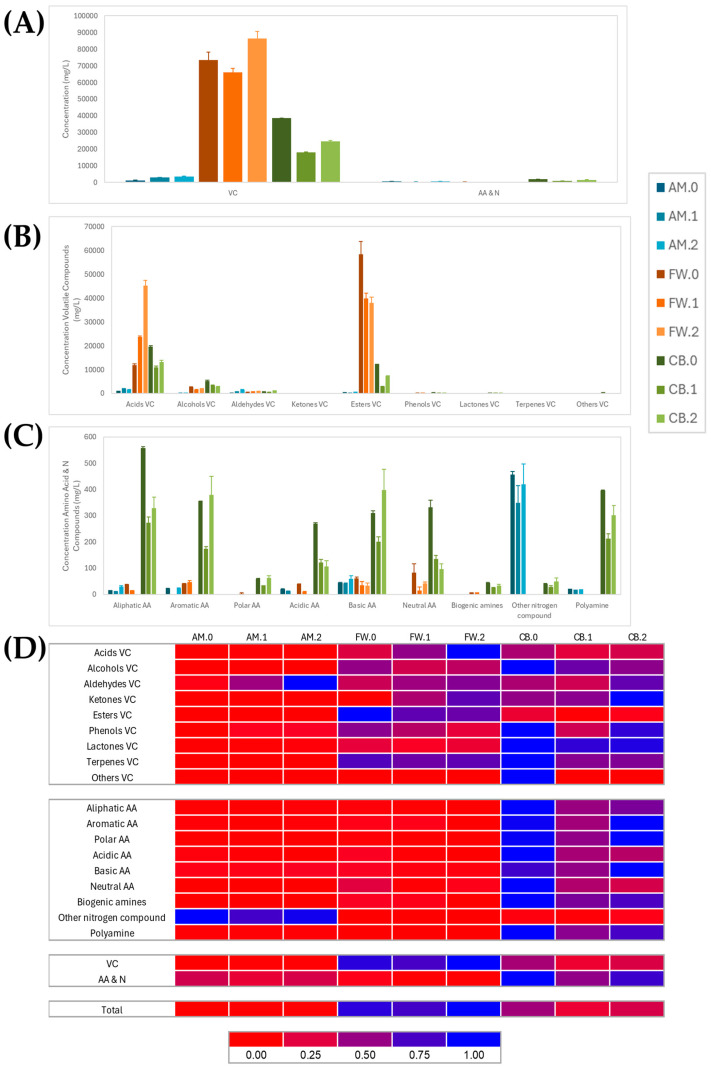
(**A**) Bar plot showing total concentration (mg/L) of metabolites including minor volatile compounds (VC), amino acids, biogenic amines, and other nitrogenous compounds (AA and N). Metabolites were also grouped into chemical families for VC (**B**) and AA and N (**C**). Bars represent mean values and their standard deviation. (**D**) Heat map showing the normalized concentration values for each chemical family. The concentration of metabolites was scaled from 0 (red) to 1 (blue). Synthetic alcohol-based medium (AM); craft beer (CB); fine wine (FW); substrates (AM.0, CB.0, and FW.0); end of loading phase (AM.1, CB.1, and FW.1); just before unloading phase (AM.2, CB.2, and FW.2).

In order to describe the metabolite profiles in quantitative terms, Figure 4 shows the metabolites grouped into chemical families, including the mean concentration values and standard deviation for each quantified metabolite in each acetification profile. The complete list of quantified metabolites can be found in Table S3 (see Supplementary Materials). Firstly, the concentration of volatile compounds (VC) was generally higher than that of the other metabolites, especially in the FW and CB profiles.

For the AM profile, most of the metabolites present were organic acids, esters, and other nitrogen compounds (ammonium ion), although the total concentration of all metabolites was significantly lower than those obtained in the other media. The FW profile exhibited a total amount of metabolites almost twice that of the CB profile; here, the final stage of the process (FW.2) presented the highest concentration of VC. This may mainly be attributed to the content of acids and esters, while the concentration of nitrogen compounds was much lower. For the CB profile, the substrate (CB.0) exhibited a higher concentration than acetification samples (CB.1 and CB.2). In this profile, it was observed that although the total concentration of metabolites was not as high as in FW, there was a higher availability of nitrogen compounds. However, the concentrations of organic acids and esters were also higher compared to the other metabolite groups. Figure 4D presents a heat map of the normalized concentration values for each chemical family. The results are generally consistent with those discussed above. While the total concentration of VC was higher in the FW profile, this was primarily due to specific families, such as acids and esters. In contrast, the distribution of both VC and nitrogen compounds was more balanced in the CB profile.

**Figure 5:**
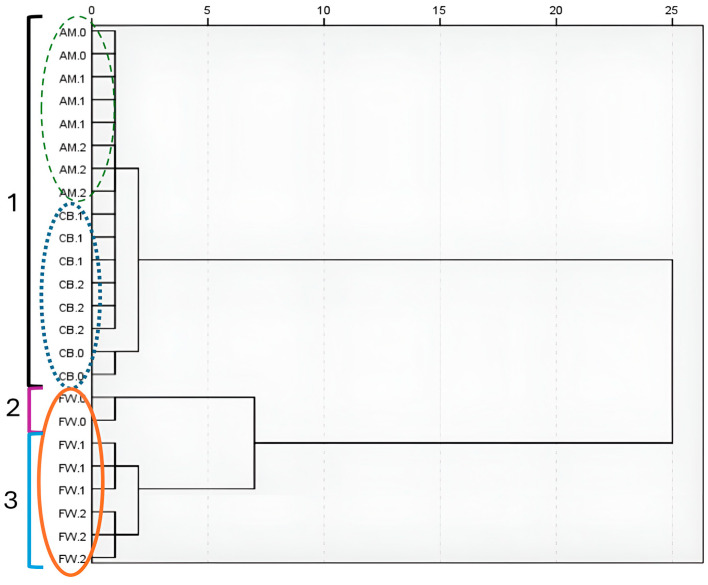
Dendrogram representing a cluster analysis applying Ward’s method for the samples of the three acetification profiles. The analysis was carried out with all studied variables at metabolomic level, including minor volatile compounds, amino acids, biogenic amines, and other nitrogenous compounds. The numbers 1 to 3 correspond to three different clusters formed at a squared Euclidean distance of < 5. Synthetic alcohol-based medium (AM); craft beer (CB); fine wine (FW); substrates (AM.0, CB.0, and FW.0); end of loading phase (AM.1, CB.1, and FW.1); just before unloading phase (AM.2, CB.2, and FW.2).

The cluster analysis represented in a dendrogram, as shown in Figure 5, allowed the classification of the samples into three groups based on the similarity of the distribution of metabolite concentrations. First, the analysis revealed that AM and CB profiles showed significant similarities between their samples, as they were grouped into the same cluster. On the other hand, the samples of the FW profile (FW.1 and FW.2) showed significant differences with both the samples of the other profiles and their raw material (FW.0).

**Figure 6:**
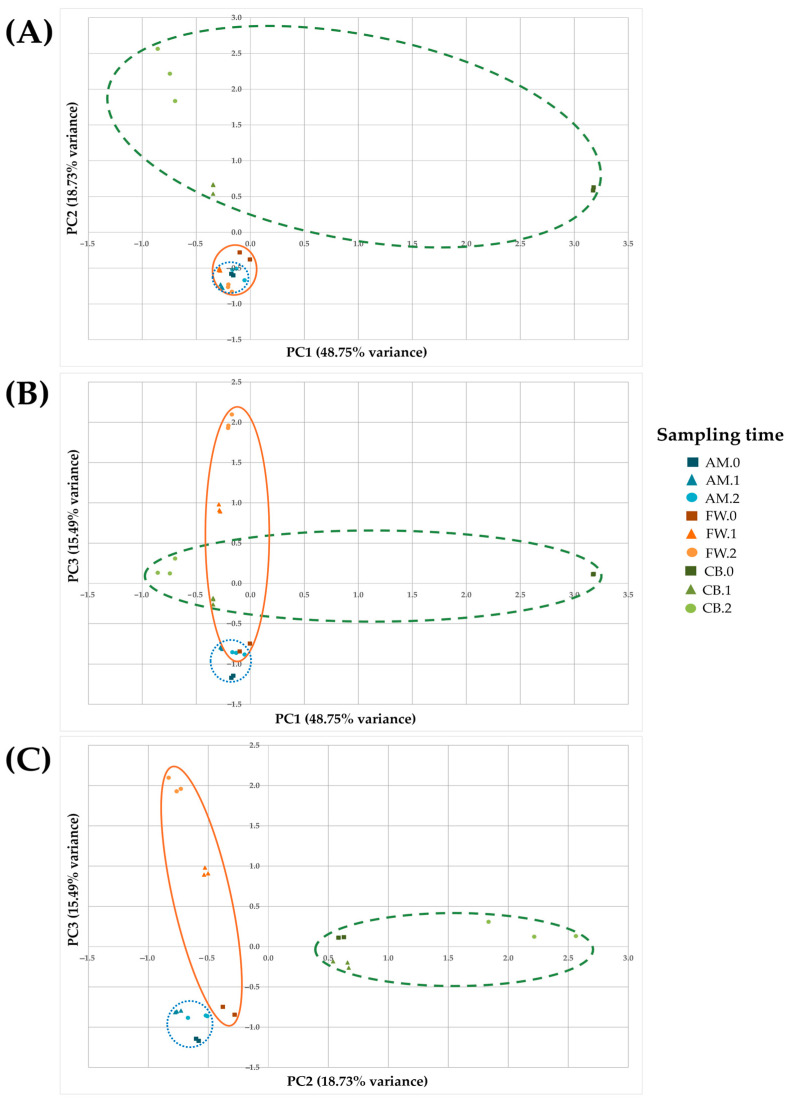
Principal component analysis (PCA) for the samples of the three acetification profiles. PCA shows three components that together account for 82.97% of the variability in the data, including minor volatile compounds, amino acids, biogenic amines, and other nitrogenous compounds. (**A**) Relationship between principal components (PC) 1 and 2; (**B**) relationship between PC1 and PC3; (**C**) relationship between PC2 and PC3. Synthetic alcohol-based medium (AM); craft beer (CB); fine wine (FW); substrates (AM.0, CB.0, and FW.0); end of loading phase (AM.1, CB.1, and FW.1); just before unloading phase (AM.2, CB.2, and FW.2).

On the other hand, a principal component analysis (PCA) was also conducted to analyze the variability of metabolite profiles (see Figure 6). The first three components accounted for 82.97% of the variance among samples of the three acetification processes, considering the 75 quantified metabolites as the selected variables. The relationship between the principal components (PC) 1 and 2 showed a significant similarity between the AM and FW profiles due to the proximity of the samples, which were consistently grouped according to the sampling time. The CB profile showed important differences during its evolution, although, in general, this relationship allowed us to differentiate the raw material (CB.0) from the rest of the samples both in beer (CB.1 and CB.2) and the other profiles (see Figure 6A). The relationship between PC1 and PC3 followed the same trend as previously described (see Figure 6B). The relationship between PC 2 and PC3, although representing a lower significance, showed a clear grouping between individual samples of each sampling time and profile, again highlighting the differences between the CB profile and the rest (see Figure 6C).

### 3.5. Correlation Analysis Between Microbiota and Metabolites in Vinegar Making

**Figure 7:**
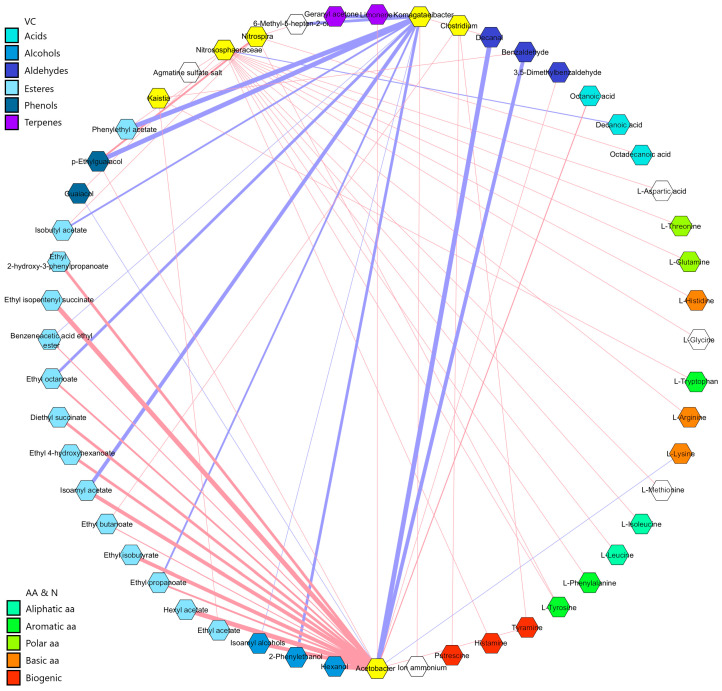
Correlation network analysis between the main microorganisms and metabolites throughout the acetification stages of the three vinegars under study by Cytoscape 3.10.2. Significant Spearman’s correlations (|ρ|> 0.48, *p* < 0.05) are shown in the diagram. Microorganisms (yellow color) and metabolites are represented as hexagons, and red and blue lines represent positive and negative correlations, respectively. The thicker the line, the higher the correlation (ranging between −1 and 1). The complete list of Spearman’s correlation values between microorganisms and metabolites can be found in Table S4 (see Supplementary Materials).

To explore the associations between the dominant microorganisms and key metabolites, Spearman’s correlation analysis was conducted and then visualized in Cytoscape (see Figure 7). A total of six microorganisms were correlated with 46 metabolites, including volatile compounds, amino acids, biogenic amines, and other nitrogenous compounds, according to established statistical criteria (|ρ|> 0.48, *p* < 0.05). It should be noted that there are additional correlations that have not been represented because they did not pass the statistical cut-off. Table S4, in the Supplementary Materials, provides the total correlations between microorganisms and metabolites. The correlation network revealed that the most abundant genus, *Komagataeibacter*, was positively correlated with the content of decanal and ammonium ion and negatively correlated with the content of twelve metabolites: two alcohols (isoamyl alcohol, 2-phenylethanol), six esters (phenylethyl acetate, isobutyl acetate, benzeneacetic acid ethyl ester, ethyl octanoate, isoamyl acetate, and ethyl propanoate), one ketone (6-methyl-5-hepten-2-one), one phenol (p-ethylguaiacol), and two terpenes (limonene and geranyl acetone). The genus *Acetobacter* exhibited positive correlations with the content of eighteen metabolites, including one acid (octanoic acid), two alcohols (isoamyl alcohols and hexanol), one aldehyde (3,5-dimethylbenzaldehyde), eleven esters (ethyl 2-hydroxy-3-phenylpropanoate, ethyl isopentenyl succinate, benzeneacetic acid ethyl ester, ethyl octanoate, diethyl succinate, ethyl 4-hydroxyhexanoate, isoamyl acetate, ethyl butanoate, ethyl isobutyrate, ethyl propanoate, and hexyl acetate), one phenol (p-ethylguaiacol), one terpene (limonene), and one biogenic amine (tyramine); meanwhile, it was negatively correlated with four metabolites: two aldehydes (decanal and benzaldehyde), one phenol (guaiacol), and one basic amino acid (L-lysine). The genera *Clostridium* and *Kaistia* were each correlated with the content of three metabolites: one ester (ethyl butanoate) and two biogenic amines (tyramine and putrescine) for *Clostridium* and one aldehyde (benzaldehyde), one ester (ethyl acetate), and one aromatic amino acid (L-tryptophan) for *Kaistia*, none of which showed negative correlations. Another bacterial genus, *Nitrospira*, showed six positive correlations and no negative associations; these were with one acid (octadecanoic acid), two esters (2-phenylethyl acetate and isobutyl acetate), one ketone (6-methyl-5-hepten-2-one), one phenol (p-ethylguaiacol), and one aromatic amino acid (L-tyrosine). Finally, the archaeal family Nitrososphaeraceae presented fourteen positive correlations with the following metabolites: one phenol (guaiacol), eleven amino acids (glycine, L-leucine, L-isoleucine, L-tyrosine, L-phenylalanine, L-threonine, L-glutamine, L-aspartic acid, L-histidine, L-arginine, and L-methionine), one biogenic amine (histamine), and one polyamine (agmatine sulfate salt), along with one negative correlation with decanoic acid.

## 4. Discussion

The acetification profiles established in this study for different substrates, namely synthetic alcohol-based medium (AM), fine wine (FW), and craft beer (CB), showed significant variability in system performance. The AM profile was not only slower but also less efficient than the rest (see Table 1). This may be attributed to the lack of nutrients typically found in natural substrates, such as organic compounds, amino acids, and vitamins, which may limit microbial activity and thus cause slower ethanol depletion and total acidity increase [3,38,39]. This latter was observed throughout the AM loading phase, where an additional discontinuous loading step was required to reach the working volume (8 L, see Figure 1). The CB profile, which was highly sugary, exhibited moderate acetification rates and, curiously, a lower increase of total acidity. This suggests that foaming problems, which reduced working volume (7.0 ± 0.2 L) together with excessive sugar content, could affect the acetification yield. Lin et al. [19] similarly noted that high sugar content in substrates could cause fermentation delays due to the need for complex carbohydrate degradation. The FW profile showed the fastest acetification cycle as well as a significantly higher acetification efficiency compared to the other media. This supports the idea that complex and rich substrates, such as wines, e.g., fine wine, can lead to an ideal environment for the growth and optimization of the oxidative activity of predominant microorganisms such as AAB [13,30,40].

Viable cell concentration revealed some distinct trends regarding efficiency. Interestingly, the AM profile showed the highest cell viability in an upward trend; the FW profile was more consistent, albeit with lower values; and the CB profile was the lowest one (see Table 1). For AM, where nutrient richness was apparently lower, AAB may be pushed to maximize their metabolic pathways as the population adapts to survive and maintain growth. Lin et al. [19] found that microbial populations can undergo succession and adaptation to environmental stress, resulting in the higher abundance of resilient strains. Moreover, the carbon sources present in CB could lead to higher competition from other microorganisms or more challenging fermentation dynamics. Trček et al. [13] highlighted the importance of microbial succession and competition during fermentation, which affects cell viability, especially in complex vinegar substrates such as apple cider. Overall, the analysis of acetification profiles showed that the composition of raw materials may influence the efficiency of vinegar production. The higher yield of FW as a substrate can be attributed to its nutrient richness, promoting microbial activity efficiency. In contrast, nutrient deficiencies, excess sugars, and foaming problems observed in AM and CB profiles illustrated challenges associated with non-optimized operating conditions, confirming findings from other studies [13,15,19].

On the other hand, the study of microbial diversity revealed a high biodiversity of the starting inoculum against acetification profiles (see Table 2 and Figure 2). Under this working system in which inoculum progressed towards different acetification profiles, microbial diversity was exposed to new substrates and conditions. In this way, the microbial communities could decrease in complexity as the system progressed towards stable acetification phases enforced by selective stress towards the predominance of the most adapted microbial groups, such as AAB, which thrive in the acidic and oxygenated environments of submerged systems [20,40,41]. The biodiversity within each profile and between them appeared to be influenced by the raw material used, as significant differences were observed in both cases (see Table 2 and Figure 2). As discussed above, the least efficient profile was also the least biodiverse despite an increasing trend during its evolution (AM). The FW and CB profiles were generally more diverse, highlighting the stability of the FW profile; the observations of Wu et al. [23] on Sichuan Baoning vinegar highlighted that more complex substrates encourage higher microbial diversity, promoting diverse metabolic activities across the process. Although the CB profile biodiversity was variable, it showed the highest phase of microbial diversity out of the three profiles in CB.2. The rich nutrient profile of CB, mainly sugars, could promote the growth of microorganisms that metabolize sugars as a carbon source, resulting in a less selective medium that could be neutralized during the loading phase by volume adjustment through foaming (CB.1) [30]. Recently, culture-dependent and culture-independent techniques have led to the detection of different AAB in spontaneously fermented sour beers [42].

Analysis of microbial dynamics revealed microbial succession patterns driven by the raw material and phase-specific operating conditions (see Figure 3). The starter inoculum showed a higher diversity, but as acetification progressed, the bacterial dominance increased with archaea decreasing to minimal levels in all profiles, consistent with metagenomic-based studies of cereal vinegar reporting bacterial abundances > 90% [19,24,43]. Most of the predominant bacteria found in acetification belonged to the family Acetobacteraceae, specifically the genera *Komagataeibacter* and *Acetobacter*. While the abundance of *Komagataeibacter* was very high and stable in the three acetification profiles, *Acetobacter*, far below *Komagataeibacter*, showed a higher abundance in the FW profile. *Komagataeibacter* has been widely found in submerged European vinegar such as spirit, wine, and fruit vinegar, as well as Asian cereal vinegar at lower abundances [13,17,40,41]. The growth characteristics of *Komagataeibacter*, such as high ethanol preference, high acetic acid production capability, and continuous oxygen demand, support its suitability to predominate throughout acetification [4,5,41]. On the other hand, *Acetobacter* might not possess the molecular adaptations necessary to thrive in a nutrient-poor medium such as AM or the ability to metabolize sugar excesses, as in CB [11,30,40]. Most molecular studies have shown that *Acetobacter* is typically harmed at acidity levels exceeding 8–10% *w*/*v*, so it is commonly found in both traditional and low-acid submerged vinegar [2,17,41]. However, at lower levels, *Acetobacter* has shown a robust ability to survive throughout the process. Regarding archaea, the family Nitrososphaeraceae was the main representative. To our knowledge, the identification of archaea in vinegar is a recent discovery that is only beginning to be studied, mainly in cereal vinegar produced by solid-state fermentation. Wu et al. [24] reported 8.06% of archaea in cereal vinegar and Han et al. [43] 0.003% by comparing four vinegar starters using metagenomics. In our study, Nitrososphaeraceae appeared to have a higher contribution in inoculum (4.97%) than in acetification samples (0.1–0.2%). The possible contribution of archaea to vinegar-making is discussed in more detail below. Other bacterial families detected at lower levels may indicate a transition towards AAB dominance with minimal non-AAB presence as conditions become more selective. Those groups able to progress and survive from inoculum to vinegar profiles, such as Rhodobacteraceae, Fusobacteriaceae, and Rhizobiales, deserve particular attention. Microbial dynamics revealed that, while the starter inoculum introduced a broad microbial community, selective pressures from acidity, nutrient-specific, and operating conditions drove microbial succession towards an AAB-dominated system, highlighting the adaptability of *Komagataeibacter* and *Acetobacter*.

Metabolite profiling analysis revealed that volatile compounds (VC) were the most abundant metabolite group, with organic acids and esters in the highest concentrations (see Figure 4). Organic acids and esters were identified as driver metabolites in Italian balsamic vinegar and Chinese Shanxi aged vinegar [18,44]. Organic acids contribute to the acidic flavor of vinegar and are usually formed during fermentation, tending to predominate at the end stage, as was the case in AM and FW profiles. Esters are associated with typical floral and fruity odors and are crucial in enhancing the organoleptic properties and complexity of vinegar and other fermented foods, such as baijiu [18,19,45]. Many esters have been found in other Spanish PDO wine vinegars, which could support the high concentration of esters found in FW [39]. The CB profile had a more balanced distribution of volatile and nitrogen compounds, although its total metabolite concentration was still lower than the FW profile. Some authors suggested that nitrogen-rich substrates may facilitate the conversion into metabolism-enhancing compounds such as esters and alcohols, thereby promoting microbial growth and flavor development in wine and Shanxi aged vinegars [17,39]. Cluster analysis showed similarities in the AM and CB profiles and a clear differentiation with the FW profile, mainly with the raw material (FW.0, see Figure 5). This suggests a unique acetification dynamic in FW, driven by its nutrient complexity and higher VC production. Traditional vinegars with longer fermentation times, studied by Wang et al. [18], showed similar metabolomic divergence to those of our work due to prolonged substrate transformations and microbial interactions that increased the accumulation of organic acids and esters critical for vinegar quality.

On the other hand, the PCA showing the sample distribution in the acetification profiles allowed us to establish a clear separation of the CB profile from the rest, mainly its substrate (CB.0, see Figure 6). Although AM and CB profiles were previously clustered together and distinguished from FW, despite their concentration differences, PCA’s ability to detect more subtle metabolite variations allowed us to reveal a higher variability of the CB profile, probably influenced by technical issues such as foaming, as mentioned in Section 3.1. It is worth noting the high availability of sugars, VC, amino acids, and other nitrogenous compounds observed in CB.0. These factors could drive microbial succession and alter metabolic pathways in CB, originating variability with other samples despite the balance of metabolites observed, in agreement with other authors [6,16,21,45]. These findings emphasize the complexity of metabolite profiling during acetification, driven by the raw material composition and fermentation dynamics.

Finally, the combined analysis of microbial succession and metabolite content outline the influence of the dominant microbiota on the organoleptic properties of vinegars. Findings obtained from the correlation of six key microbial groups with 46 metabolites highlight the differential metabolic contributions of specific taxa (see Figure 7). *Komagataeibacter* has a preference for the incomplete oxidation of ethanol into acetic acid; although no significant positive correlations with acids were found here, as Zhu et al. [17] reported in Shanxi aged vinegar, those with ammonium ion and decanal might be indicative of both consistent assimilation of nitrogen sources and the promotion of aldehyde-based metabolic pathways to maintain the membrane stability as strategies against high-acid stress [18,46,47]. Negative correlations with esters (e.g., 2-phenylethyl acetate and isoamyl acetate) and alcohols (e.g., isoamyl alcohol), among others, might also indicate that *Komagataeibacter*, despite its abundance, is not involved in the biosynthesis of metabolites but rather in their consumption. Moreover, *Acetobacter* was positively correlated with many of these metabolites, including esters, alcohols, and acids, among others. *Acetobacter* spp. have been identified as primary contributors to the variation of volatile flavor compounds in Chinese vinegar [18,43,48]. Considering that *Acetobacter* was mainly found in FW samples, it could be hypothesized that this genus influenced the higher organoleptic complexity shown by this profile. The high number of positive correlations of *Acetobacter* with esters (11) may lead to microbial esterification pathways between alcohols and acids, which was also shown, contributing to the sensory improvement of FW vinegar [18,19,39]. These findings have revealed that *Acetobacter* and *Komagataeibacter* may use different metabolic strategies of generation and consumption, respectively, of key compounds, leading to a cycle of cooperation and mutual support that, to our knowledge, had not been shown until now. This would also explain the *Komagataeibacter* abundance, which would focus on VC consumption and efficient oxidative metabolism to obtain a competitive advantage.

The bacterial genera *Kaistia* and *Nitrospira* have not been previously reported in this medium to our knowledge since their natural environments are usually soil and aquatic ecosystems, playing a role in nitrogen cycles [49,50,51]. Their identification in vinegar may represent a new niche for these genera, and due to the positive correlations shown with esters and aromatic amino acids, they might contribute secondarily to the biosynthesis of aromatic and flavor precursors from available nitrogen sources, complementing the role of AAB [17]. The presence of biogenic amines in fermented foods, particularly those with high content of amino acids and proteins, such as yogurt, cheese, wine, vinegar, fermented sausages, and fermented fish, has been the subject of considerable research. These compounds, produced by the action of decarboxylases on free amino acids [52], have been previously identified in wines and vinegars of diverse origins in Europe [53,54]. While the relationship between certain yeast and lactic acid bacteria species and the formation of biogenic amines has been previously described, their presence in vinegar remains unclear [55]. However, our analysis has enabled the interrelation of the presence of *Clostridium* with putrescine and tyramine in vinegar, suggesting a potential origin (see Figure 7). The presence of *Clostridium sensu stricto I* has been linked to the formation of tyramine, histamine, and cadaverine in fermented sausages, while *Clostridium aceticum* has been observed to produce putrescine, cadaverine, and agmatine in acidic media [56,57]. *Nitrososphaeraceae* was consistent throughout acetification but mostly in inoculum samples. Before the present study, the presence of archaea in vinegar was scarcely reported in traditional [24,43] and submerged vinegar [11], but their role has not yet been clarified. Note that Nitrososphaeraceae was positively correlated with several amino acids (11), such as L-tyrosine, L-leucine, and L-isoleucine, as well as biogenic amines like histamine. Considering this and that its metabolism has been linked to ammonia-oxidizing pathways, intermediates such as nitrite and nitrate could be provided as esterification precursors and other key secondary pathways, thus indirectly contributing to the metabolomic profiling of vinegars [47,58]. Overall, the correlation analysis between the dominant microbiota and key metabolites has allowed the establishment of previously unknown functional associations for vinegar production in Europe.

## 5. Conclusions

In this study, the acetification profiles of three vinegars were characterized and compared based on microbial succession and its correlation with the key metabolome under operating conditions typical of Europe. The technical analysis highlighted the importance of working with optimized operating conditions as well as the composition of the acetification raw material used. The combined application of metagenomic and metabolomic tools allowed the definition of microbial dynamics led by AAB, mainly *Komagataeibacter* and *Acetobacter*, and other microbial groups, some of which had never been found in vinegar before, contributing secondarily to the organoleptic profile of the final products. Furthermore, previously unknown functional associations between microorganisms and metabolites were clarified, such as the simultaneous positive and negative correlations of *Acetobacter* and *Komagataeibacter* with the same compounds, suggesting a cooperation cycle and mutual support, thus defining both their abundance and the organoleptic properties of vinegars. To the best of our knowledge, there is no research background implementing a multi-omic strategy to characterize European vinegar at the level of detail intended here. Although further research is necessary, the novel findings of this study can contribute to the improvement of existing knowledge and help the vinegar industry to establish the basis for optimizing operating conditions and designing safe vinegars with organoleptic properties demanded by consumers.

## Figures and Tables

**Table 1:** Main system variables of the three acetification profiles. Data show the mean values at the sampling times and their standard deviation (SD). Variables employed to obtain the acetification efficiency of each profile (*r_A_* and *p_A_*) are also detailed.

Variable (Mean ± SD)	AM.0	AM.1	AM.2	CB.0	CB.1	CB.2	FW.0	FW.1	FW.2
Cycle time (h)	-	8.8 ± 2.8	28.9 ± 2.6	-	2.8 ± 0.4	24.3 ± 1.1	-	3.0 ± 0.1	21.4 ± 0.1
Volume (L)	-	8.0 ± 0.1	8.0 ± 0.1	-	7.0 ± 0.2	7.0 ± 0.2	-	8.0 ± 0.1	8.0 ± 0.1
Ethanol (% *v*/*v*)	10.0 ± 0.3	5.0 ± 0.1	1.1 ± 0.2	9.5 ± 0.3	4.7 ± 0.2	1.2 ± 0.1	9.8 ± 0.3	4.9 ± 0.1	1.3 ± 0.3
Total acidity (% *w*/*v*)	0.1 ± 0.1	4.5 ± 0.2	7.2 ± 0.1	0.2 ± 0.1	4.2 ± 0.4	6.8 ± 0.7	0.2 ± 0.1	4.3 ± 0.1	7.9 ± 0.2
Viable cell (10^8^ cell/mL)	-	1.69 ± 0.34	2.30 ± 0.29	-	0.84 ± 0.70	1.05 ± 0.70	-	1.43 ± 0.33	1.47 ± 0.28
		**AM**			**CB**			**FW**	
Mean acetification rate (*r_A_*) [g acetic acid/(L h)]		1.3 ± 0.1			1.6 ± 0.1			1.9 ± 0.1	
					
Global acidity production (*p_A_*) (g acetic acid/h)		10.2 ± 1.0			11.3 ± 0.5			15.2 ± 0.5	
					

Synthetic alcohol-based medium (AM); craft beer (CB); fine wine (FW); substrates (AM.0, CB.0, and FW.0); end of loading phase (AM.1, CB.1, and FW.1); just before unloading phase (AM.2, CB.2, and FW.2).

**Table 2:** Alpha diversity indexes for quantification of the biodiversity degree of both starting inoculum and the three acetification profiles. Data show the mean values at the sampling times and their standard deviation (SD).

Sample	Observed Features	Shannon	Simpson
Inoculum	2417 ± 784	3.40 ± 1.30	0.434 ± 0.154
AM.1	198 ± 56	0.27 ± 0.10	0.042 ± 0.016
AM.2	294 ± 52	0.36 ± 0.04	0.054 ± 0.006
CB.1	272 ± 22	0.37 ± 0.04	0.056 ± 0.007
CB.2	720 ± 197	0.73 ± 0.20	0.105 ± 0.029
FW.1	450 ± 138	0.61 ± 0.19	0.104 ± 0.025
FW.2	359 ± 158	0.50 ± 0.21	0.079 ± 0.029

Synthetic alcohol-based medium (AM); craft beer (CB); fine wine (FW); substrates (AM.0, CB.0, and FW.0); end of loading phase (AM.1, CB.1, and FW.1); just before unloading phase (AM.2, CB.2, and FW.2).

## Data Availability

The 16S rRNA sequencing raw data obtained in this study are deposited in the Sequence Read Archive (SRA) repository (NCBI), accession number: PRJNA891065 (https://www.ncbi.nlm.nih.gov/sra/).

## Supplementary Materials

The following supporting information can be downloaded at https://www.mdpi.com/article/10.3390/foods14010056/s1. Table S1: Reads obtained from samples by applying Illumina amplicon sequencing and amplicon sequence variants (ASVs) resulting after filtering, denoising, merging, and chimera filtering procedures. AM, synthetic alcohol-based medium; CB, craft beer; FW, fine wine. End of the loading phase (AM.1, CB.1, and FW.1); just before the unloading phase (AM.2, CB.2, and FW.2); Table S2: List of the total identified OTUs and the taxon assigned to each of them up to a genus level as a maximum specificity. The consensus level of each assignment is included; Table S3: Identified metabolites, including volatile compounds, amino acids, other nitrogen compounds, biogenic amines, and polyamines, and their concentration (mg/L) in the samples of synthetic alcohol-based medium (AM), fine wine (FW), and craft beer (CB). Raw material (AM.0, FW.0, and CB.0), end of the loading phase (AM.1, FW.1, and CB.1), and just before unloading phase (AM.2, FW.2, and CB.2); Table S4: Spearman’s correlation analysis (*p* < 0.05) conducted between metabolites present in the samples of the acetification profiles (AM.1, FW.1, CB.1, AM.2, FW.2, and CB.2) and the 10 most abundant microorganisms.
