# Supplementary material for: Correlating Microbial Dynamics with Key Metabolomic Profiles in Three Submerged Culture-Produced Vinegars
Source: Foods. 2024 Dec 28;14(1):56. doi: 10.3390/foods14010056 (PMC11720010; doi:10.3390/foods14010056)
Supplement: Supplementary file 1 [file foods-14-00056-s001.zip › Table S1.pdf]

Table S1. Reads obtained from samples by applying Illumina amplicon sequencing and amplicon sequence variants (ASVs) resulting after filtering, denoising, merging, and chimera filtering procedures. AM, synthetic alcohol-based medium; CB, craft beer; FW, fine wine. End of the loading phase (AM.1, CB.1, FW.1); just before the unloading phase (AM.2, CB.2, FW.2).

Sample	1. Reads	2. Filtering	3. Denoising	4. Merging	5. Chimera filtering
Inoculum.1	219,463	209,788 (95.6%)	204,428 (93.1%)	193,516 (88.2%)	187,454 (85.4%)
Inoculum.2	211,731	202,638 (95.7%)	197,197 (93.1%)	186,220 (88.0%)	179,895 (85.0%)
AM.1_1	212,847	202,637 (95.2%)	201,873 (94.8%)	200,811 (94.4%)	198,771 (93.4%)
AM.1_2	211,649	201,729 (95.3%)	200,929 (94.9%)	200,123 (94.6%)	199,466 (94.2%)
AM.1_3	205,109	194,764 (95.0%)	193,828 (94.5%)	191,927 (93.6%)	190,002 (92.6%)
AM.2_1	206,885	197,304 (95.4%)	196,480 (95.0%)	195,250 (94.4%)	193,867 (93.7%)
AM.2_2	208,403	198,466 (95.2%)	197,177 (94.6%)	195,569 (93.8%)	194,353 (93.3%)
AM.2_3	219,790	209,353 (95.3%)	207,960 (94.6%)	206,275 (93.9%)	204,711 (93.1%)
CB.1_1	213,290	203,004 (95.2%)	202,011 (94.7%)	200,351 (93.9%)	198,187 (92.9%)
CB.1_2	202,849	193,643 (95.5%)	192,608 (95.0%)	190,958 (94.1%)	189,512 (93.4%)
CB.1_3	208,086	198,927 (95.6%)	197,997 (95.2%)	196,818 (94.6%)	196,010 (94.2%)
CB.2_1	213,583	203,773 (95.4%)	202,409 (94.8%)	200,085 (93.7%)	198,184 (92.8%)
CB.2_2	214,472	204,834 (95.5%)	202,152 (94.3%)	197,470 (92.1%)	194,760 (90.8%)
CB.2_3	207,689	197,863 (95.3%)	195,455 (94.1%)	191,558 (92.2%)	188,744 (90.9%)
FW.1_1	218,750	208,264 (95.2%)	206,276 (94.3%)	202,816 (92.7%)	197,451 (90.3%)
FW.1_2	218,473	208,391 (95.4%)	206,949 (94.7%)	205,107 (93.9%)	200,021 (91.6%)
FW.1_3	211,723	201,522 (95.2%)	200,549 (94.7%)	199,237 (94.1%)	194,768 (92.0%)
FW.2_1	218,671	207,879 (95.1%)	206,088 (94.2%)	202,789 (92.7%)	198,847 (90.9%)
FW.2_2	207,785	198,366 (95.5%)	197,352 (95.0%)	196,081 (94.4%)	193,134 (93.0%)
FW.2_3	217,756	207,336 (95.2%)	206,397 (94.8%)	204,894 (94.1%)	202,957 (93.2%)
