# Supplementary material for: Correlating Microbial Dynamics with Key Metabolomic Profiles in Three Submerged Culture-Produced Vinegars
Source: Foods. 2024 Dec 28;14(1):56. doi: 10.3390/foods14010056 (PMC11720010; doi:10.3390/foods14010056)
Supplement: Supplementary file 1 [file foods-14-00056-s001.zip › Table S3.pdf]

Table S3. Identified metabolites, including volatile compounds, amino acids, other nitrogen compounds, biogenic amines, and polyamines, and their concentration (mg/L) in the samples of synthetic alcohol-based medium (AM), fine wine (FW), and craft beer (CB). Raw material (AM.0, FW.0, CB.0), end of the loading phase (AM.1, FW.1, CB.1), and just before unloading phase (AM.2, FW.2, CB.2).

		Metabolite (mg/L)	AM.0	AM.1	AM.2	FW.0	FW.1	FW.2	CB.0	CB.1	CB.2
Volatile compounds	Acids	Pentanoic acid	nd	547.6±51.5	428.7±23.3	nd	15181.9±710.4	34464.1±2557.9	952.5±78.3	4659.2±241.6	8562.4±523.3
		Hexanoic acid	nd	215.3±10.7	nd	1116.9±101.8	1412.4±69.2	2121.9±129.4	1845.3±74.5	1135.1±57.6	1214.2±80.2
		Octanoic acid	nd	490.2±9.2	495.5±35.9	10082.6±577.5	6428.5±303	7577.4±420.4	13268.5±423.3	3638.8±323.1	2066±150.3
		Decanoic acid	nd	181.2±12.1	159.6±15.6	164.9±11.2	172.9±12	203.1±8.9	1018.9±62.3	151.7±10.1	136.5±9.5
		Dodecanoic acid	30.9±2.1	73.9±5.3	51±2.7	36.6±3	53.9±4.9	75.7±6.8	89.2±3.3	70.6±7	61.7±1.1
		Tetradecanoic acid	176.5±10.6	143.5±10.2	109.3±8.5	79.4±3.4	121.2±9.6	182.5±4.7	221.3±7	206±17.9	160.1±10.3
		Hexadecanoic acid	608±33.9	302.2±22	298±13.6	324.2±11.3	311.8±27.8	598±44	2241.6±46.8	1103.1±46.9	902.2±25.9
		Octadecanoic acid	nd	nd	6.7±0.5	nd	nd	28±0.9	41.7±3.3	21.6±2	50.7±3.8
	Alcohols	Isoamyl alcohols	nd	nd	nd	407.8±29.8	160.2±13.4	83.8±6.8	178±4.8	33.9±1.8	14.1±1.4
		2,3-Butanediol	nd	nd	nd	nd	nd	nd	583.5±31	127.8±6.2	nd
		Furfuryl alcohol	nd	nd	nd	755.6±61.6	797.3±42.6	1560.7±115.9	1562.9±110.9	2081.3±153.9	1681.2±147
		Hexanol	nd	nd	nd	1163.4±75.7	365.3±1.9	nd	nd	nd	nd
		2-Phenylethanol	nd	nd	nd	75.3±3.8	47.1±1.4	50.3±3.1	103.1±7	39.1±2.8	43.4±3.8
		4-Vinylphenol	nd	87.6±7.6	69.9±3.3	149.6±49.1	140.3±13.9	199.3±174.1	866.5±71	380.3±23.3	398.7±4.7
		2-Methoxy-4-vinylphenol	nd	nd	nd	81.3±52.9	45.2±2.2	51.9±3	1917±64.2	680.8±61.5	626.6±6.9
	Aldehydes	Benzaldehyde	nd	310.5±24.2	911.6±91.1	39.1±7.8	37±2.9	82.8±2.1	35.7±1.9	221.9±22.1	558.1±36
		Phenylacetaldehyde	nd	nd	nd	14±2.3	7.1±0.5	10.6±0.7	163.7±16.1	49.3±3.7	50.9±3.4
		Octanal	nd	nd	nd	0.8±0.1	nd	nd	3.8±0.3	nd	nd
		Decanal	nd	2.5±0.2	1.7±0.2	1.3±0.3	0.6±0	1.8±0.1	1.7±0.1	3.3±0.1	1.8±0.1
		3,5-Dimethylbenzaldehyde	146±3.2	404.5±23.8	594.1±40.8	444.2±1.1	677.1±48	743.6±60.2	442.3±30.7	185.9±16.4	404±15.8
	Ketones	6-Methyl-5-hepten-2-one	nd	nd	nd	nd	9.6±0.2	15.9±0.7	11.7±1.1	12.4±1.2	23.1±1.7
	Esthers	Ethyl acetate	nd	nd	156.4±9.7	64.7±4.8	43.8±2.8	50.3±3.8	11.3±0.4	29.5±1.6	32.2±3.1
		Isobutyl acetate	nd	2±0.1	5.3±0.4	5.2±0.5	145.4±12.3	269.9±21.6	48.6±2.1	157.9±2.3	256.3±13.3
		Hexyl acetate	nd	nd	nd	nd	15.6±1.1	nd	nd	nd	nd
		2-Phenylethyl acetate	nd	3.1±0.1	16±1.1	171.6±2.5	1652.7±164	2706.4±255	2548.6±171.9	1747.5±160.1	3309.6±223.6
		Ethyl propanoate	nd	nd	nd	228.8±15.4	175.3±10.5	140.7±8.1	119.9±8.7	96.3±7.7	81.9±7.9
		Ethyl isobutyrate	nd	nd	nd	15.3±0.7	21.1±0.2	19.5±1.3	1.3±0.1	2.8±0.1	2.6±0.1

		Ethyl butanoate	238.5±0.8	37.2±2.8	34±2.6	348.5±30.8	93.3±1.9	55.9±5	684.4±26.3	87.3±7.3	32.5±1
		Ethyl pentanoate	12±0.3	nd	nd	nd	nd	nd	59.6±3.1	nd	nd
		Ethyl hexanoate	nd	37.9±3	46.4±4	1330±115.8	7829.1±759.2	5311.7±345.5	6114.2±188.6	nd	2529.8±6.2
		Isoamyl acetate	nd	nd	nd	363.6±34.8	nd	nd	728.6±12.7	nd	nd
		Ethyl cis-4-hexenoate	nd	nd	nd	nd	nd	nd	190±3.9	10.8±0.1	nd
		Ethyl 4-hydroxybutanoate	nd	nd	nd	7.9±1.4	3.1±0.3	2.1±0.1	31.8±1.9	7.9±0.2	7.1±0.4
		Ethyl 4-hydroxyhexanoate	nd	nd	nd	19±0.7	13.2±0.9	14.4±1.3	nd	nd	nd
		Ethyl benzoate	18.7±0.2	18.2±1.3	26.3±0.5	28.9±5.1	19.7±1.1	19.1±1.6	265.5±23.6	53.6±3.9	42.1±2.6
		Diethyl succinate	nd	nd	nd	51843.9±4898.5	28015.4±1315	28096.7±2294.5	361.3±5.8	247±17	302.9±21.2
		Ethyl octanoate	1.7±0.1	1.7±0.1	1.7±0.1	56.6±0.6	4.6±0.2	4±0.3	254±2.6	3.2±0.4	3.2±0.2
		Benzeneacetic acid ethyl ester	10.0±0.9	114.4±10.3	161.7±14.8	210.9±8.7	848.5±38.1	946.8±92.3	336.9±24.6	446.9±98.6	463.4±34.5
		Ethyl benzenepropanoate	nd	nd	nd	nd	nd	6.7±2.2	330.6±14.8	50.7±2.2	33.3±2.1
		Ethyl isopentenyl succinate	nd	nd	nd	3476.4±260	835.6±10.1	330.7±8.7	26.8±1.5	22±1.1	8±0.6
		Ethyl 2-hydroxy-3-phenylpropanoate	nd	nd	nd	155.1±2.1	96.7±9.2	105.3±4.9	26.4±4.2	nd	13.3±0.8
		Trans-Methyldihydrojasmonate	nd	5.4±0.2	8.4±0.8	6.1±0.2	5.7±0.5	6.5±0.4	10.6±0.8	10.9±1	8.7±0.8
	Phenols	Guaiacol	nd	38±2.4	42.8±3.6	nd	23.9±2.1	60.4±2.7	39.4±4.9	83±3.1	129.5±5
		p-Ethylguaiacol	nd	nd	nd	146±24.5	81.1±3.6	nd	229.1±13.8	nd	92.8±8.6
	Lactones	5-Valerolactone	nd	nd	9.4±16.4	36±7	22.6±1.2	33.3±1.7	23.3±0.6	49±2.3	42.9±2.7
		Gamma-nonalactone	5.7±0.5	6±0.2	6.1±0.3	18.1±0.5	14.4±1.2	16.3±1.2	211.2±7.7	146.7±8.5	161.2±10
	Terpenes	Limonene	nd	nd	nd	22.2±1.4	25.2±2.3	26.9±0.2	23.3±0.1	20.3±1.6	20.1±1.4
		Nerol	nd	nd	nd	6.5±2.4	nd	nd	20.8±1	nd	nd
		Geranyl acetone	nd	nd	nd	12.5±0.7	11.3±0.2	12.3±1	13.6±1.2	11.5±0.6	13±0.6
	Others	2,5,5-Trimethyl-2,6-heptanedione	nd	nd	nd	nd	nd	nd	279±1.1	nd	nd
**Amino acids (aa)**	Aliphatic aa	Glycine	nd	nd	nd	5.6±0.5	nd	nd	83±0.1	38.9±3.9	63±16.4
		L-Alanine	14.4±0.3	nd	nd	15.2±0	8.3±1	nd	255.1±1.1	116.2±8.8	98.7±11.4
		L-Leucine	nd	11.1±0.5	17.9±1.9	10.5±0	6.6±1.3	nd	147.2±2.4	75.4±6.5	105.6±12.2
		L-Isoleucine	nd	nd	13.1±0.3	nd	nd	nd	72.9±1.3	42.1±2.5	60.7±5.1
		L-Norleucine	nd	nd	nd	6.6±0	nd	nd	nd	nd	nd
	Aromatic aa	L-Tyrosine	nd	nd	nd	41.7±0	46.5±5.8	nd	168.5±0.8	96±4.7	213.8±38.9
		L-Phenylalanine	nd	nd	nd	nd	nd	nd	138.6±0.2	77.4±4.6	165.6±31.2
		L-Tryptophan	23.9±0.1	nd	24±1.2	nd	nd	nd	47.8±0.3	nd	nd
	Polar aa	L-Threonine	nd	nd	nd	2.4±3.4	nd	nd	15.8±3	11.2±0.8	24.2±4.3
		L-Glutamine	nd	nd	nd	nd	nd	nd	43.3±1.3	21±1.4	38±3.8
	Acidic aa	L-Aspartic acid	nd	nd	nd	19.3±0.9	nd	nd	116.1±4	42.1±4.7	43.2±8.8

	L-Glutamic acid	20.5±1.7	13.1±0.6	nd	21.3±1	10.8±0.8	nd	138±0.2	70.7±6.4	57.6±8.5
	L-Ornithine	nd	nd	nd	nd	nd	nd	15.9±0.2	9.1±0.5	5.8±5.1
Basic aa	L-Histidine	nd	nd	nd	11.5±1.5	nd	nd	59.3±3	35±4.3	110.6±36.1
	L-Lysine	22.1±0.5	20.4±0.6	30.4±16.4	23.4±0	11.7±10.1	34.1±8.9	65.1±7.1	44.1±5	21.3±5.5
	L-Arginine	22.9±0.2	21.4±1.1	28.4±24.6	26.1±2.5	23.2±3.6	nd	184.7±0.7	121.4±9.1	265.1±44.3
Neutral aa	L-Methionine	nd	nd	nd	nd	nd	nd	13.1±0.3	9±0.8	15.6±4.9
	L-Proline	nd	nd	nd	82.9±32.6	14.2±15	41.4±5	103.2±21.7	15.4±13.8	nd
	L-Citrulline	nd	nd	nd	nd	nd	nd	10.6±2.4	nd	nd
	Gamma-aminobutyric acid	nd	nd	nd	nd	nd	nd	205.4±2.6	110.4±5.5	79.9±16.1
**Biogenic amines**	Histamine	nd	nd	nd	nd	nd	nd	34.6±0.1	20.4±1	30.5±4.7
	Tyramine	nd	nd	nd	1.4±0	1.4±0	nd	2.6±0	1.3±0.2	nd
	Putrescine	nd	nd	nd	5.3±0	3.8±1	nd	7.3±0.1	4.4±0.2	2.4±2.1
**Other nitrogen compound**	Ion ammonium	456±13	349.3±64.8	419.7±76.7	nd	nd	nd	39.9±1.4	29.2±4.3	49.2±13.6
**Polyamine**	Agmatine sulfate salt	19.4±0.8	15.1±1.9	17.9±0.8	nd	nd	nd	395.1±2.3	212.8±17.1	301.9±36.7
