# Supplementary material for: Correlating Microbial Dynamics with Key Metabolomic Profiles in Three Submerged Culture-Produced Vinegars
Source: Foods. 2024 Dec 28;14(1):56. doi: 10.3390/foods14010056 (PMC11720010; doi:10.3390/foods14010056)
Supplement: Supplementary file 1 [file foods-14-00056-s001.zip › Table S4.pdf]

Table S4. Spearman's correlation analysis ($p < 0.05$) conducted between metabolites present in the samples of the acetification profiles (AM.1, FW.1, CB.1, AM.2, FW.2, CB.2) and the 10 most abundant microorganisms.

**Metabolite**	***Acetobacter***	***Bacillus***	***Cetobacterium***	***Clostridium***	***Kaistia***	***Komagataeibacter***	***Nitrososphaeraceae***	***Nitrospira***	***Rhodobacter***	***Sphingomonas***
Pentanoic acid	0.3897	-0.3455	-0.1643	-0.0762	-0.1813	-0.3225	-0.0973	0.2327	-0.1168	0.2499
Hexanoic acid	0.4280	-0.4255	-0.0441	0.0742	-0.2423	-0.4525	0.0096	0.3468	0.0575	0.0485
Octanoic acid	0.6594	-0.4240	-0.0855	0.1559	-0.2642	-0.3739	-0.1816	0.1795	0.0120	0.0546
Decanoic acid	0.2986	-0.1185	-0.1660	-0.2082	-0.3740	0.1914	-0.5216	-0.3053	-0.1828	0.3465
Dodecanoic acid	-0.3027	-0.1711	0.0919	-0.3372	-0.4230	0.3447	-0.1577	-0.3037	-0.2357	0.3591
Tetradecanoic acid	-0.2989	-0.3853	0.2758	-0.0223	-0.2315	0.0863	0.0478	-0.1437	0.0799	0.1214
Hexadecanoic acid	-0.3774	-0.3518	0.3858	0.1669	-0.0456	-0.1457	0.2775	0.1692	0.2519	-0.1467
Octadecanoic acid	-0.3717	-0.2138	0.1252	-0.1069	0.1422	-0.3583	0.4698	0.5109	0.1419	-0.0840
Isoamyl alcohols	0.9135	-0.1796	-0.2465	0.3575	-0.2429	-0.4821	-0.1778	0.2511	0.0912	-0.0234
2,3-Butanediol	-0.2425	-0.3773	0.5109	0.3047	-0.1524	0.2353	-0.1342	-0.3437	0.2023	-0.1586
Furfury alcohol	-0.0366	-0.5096	0.2764	0.1482	-0.1667	-0.2853	0.1742	0.2503	0.1779	-0.0793
Hexanol	0.9455	0.0199	-0.3029	0.3700	-0.1895	-0.4162	-0.1159	0.2130	0.1183	-0.1419
2-Phenylethanol	0.4585	-0.4096	-0.0238	0.2309	-0.2081	-0.5460	0.1113	0.4271	0.1993	-0.0524
4-Vinylphenol	-0.2182	-0.1923	0.2248	0.1921	0.0777	-0.3879	0.3428	0.4088	0.3506	-0.3005
2-Methoxy-4-vinylphenol	-0.3411	-0.2317	0.3209	0.1952	0.0001	-0.1692	0.3542	0.2162	0.3169	-0.2464
Benzaldehyde	-0.5748	0.2247	0.1884	-0.1662	0.6005	0.1671	0.3042	-0.0044	-0.0317	-0.1758
Phenylacetaldehyde	-0.2949	-0.2359	0.2813	0.1868	-0.0079	-0.2451	0.4088	0.2932	0.3136	-0.2307
Decanal	-0.7352	-0.0725	0.4225	-0.0563	-0.0860	0.4969	-0.0678	-0.4193	0.0430	0.0103
3,5-Dimethylbenzaldehyde	0.5365	-0.0351	-0.2744	-0.1376	0.0659	-0.2381	-0.1313	0.1900	-0.2464	0.1387
6-Methyl-5-hepten-2-one	0.0531	-0.3000	0.0214	0.0593	-0.0479	-0.5483	0.4037	0.5923	0.1770	-0.0907
Ethyl acetate	-0.0883	-0.1252	0.2375	0.0123	0.4924	0.1114	-0.0297	-0.1051	-0.0391	-0.2122
Isobutyl acetate	0.1573	-0.3567	-0.0329	0.0609	-0.0992	-0.5402	0.2952	0.5229	0.1184	0.0317
Hexyl acetate	0.9461	0.0093	-0.2901	0.3778	-0.1889	-0.4136	-0.1137	0.1958	0.1366	-0.1422
2-Phenylethyl acetate	0.1257	-0.2977	-0.0465	0.0766	-0.0629	-0.5910	0.3777	0.6026	0.1565	-0.0179
Ethyl propanoate	0.7205	-0.3516	-0.1139	0.3209	-0.2523	-0.5414	-0.0538	0.3524	0.1612	-0.0477
Ethyl isobutyrate	0.8114	-0.2586	-0.2612	0.1480	-0.2342	-0.4353	-0.1759	0.2397	-0.0294	0.0923
Ethyl butanoate	0.6409	-0.3217	0.0867	0.5155	-0.3102	-0.1927	-0.2968	-0.0899	0.2400	-0.1466
Isoamyl acetate	0.8483	-0.1205	-0.3481	0.2096	-0.1854	-0.5784	-0.0249	0.4310	0.0414	0.0293
Ethyl cis-4-hexenoate	-0.2428	-0.3827	0.5200	0.3019	-0.1563	0.2358	-0.1332	-0.3416	0.1956	-0.1589
Ethyl 4-hydroxybutanoate	-0.0651	-0.3410	0.2821	0.2579	-0.0977	-0.2891	0.2863	0.2750	0.3259	-0.2614
Ethyl 4-hydroxyhexanoate	0.7644	-0.2650	-0.2335	0.0457	-0.2335	-0.3298	-0.2192	0.1620	-0.1088	0.1109

Ethyl benzoate	-0.4170	-0.2384	0.4473	0.2743	0.1164	-0.0575	0.2749	0.0716	0.3385	-0.3288
Diethyl succinate	0.7774	-0.2203	-0.2834	0.1206	-0.2149	-0.3934	-0.2169	0.2013	-0.0608	0.1459
Ethyl octanoate	0.7347	-0.3562	-0.1262	0.2415	-0.2495	-0.5443	-0.0155	0.3809	0.1229	-0.0648
Benzeneacetic acid ethyl ester	0.6548	-0.3647	-0.1897	0.1588	-0.1987	-0.4955	-0.0357	0.3109	0.0495	0.0824
Ethyl benzenpropanoate	-0.3785	-0.3313	0.4235	0.1902	-0.0617	-0.0315	0.2225	0.0532	0.2747	-0.1993
Ethyl isopentenyl succinate	0.9682	-0.1205	-0.3028	0.2798	-0.2365	-0.4333	-0.1787	0.2146	0.0378	-0.0286
Ethyl 2-hydroxy-3-phenylpropanoate	0.7620	-0.2623	-0.2873	0.0269	-0.2222	-0.3955	-0.1628	0.2288	-0.0945	0.1418
Trans-Methyldihydrojamonate	-0.4707	-0.4011	0.5347	0.1388	0.2047	0.0862	0.1650	-0.0219	0.1881	-0.3364
Guaiacol	-0.4913	-0.1329	0.1939	-0.0692	0.1549	-0.2515	0.5026	0.4221	0.1989	-0.1610
p-Ethylguaiacol	0.4912	0.1212	-0.3079	0.1707	-0.0027	-0.6466	0.4411	0.6686	0.2350	-0.2366
5-Valerolactone	-0.0429	-0.3966	0.2998	0.1745	0.1296	-0.3746	0.3084	0.3166	0.1534	-0.1745
Gamma-nonalactone	-0.3499	-0.2027	0.2907	0.1541	0.0177	-0.1975	0.3869	0.2717	0.3172	-0.2441
Limonene	0.5220	-0.4599	0.0007	0.1967	-0.2486	-0.5126	0.1238	0.3725	0.1287	-0.0570
Geranyl acetone	0.3520	-0.4019	0.0620	0.2341	-0.1740	-0.5515	0.2367	0.4667	0.2220	-0.1404
Glycine	-0.3631	-0.0915	0.2292	0.0586	0.0446	-0.2112	0.4901	0.3342	0.3162	-0.2342
L-Alanine	-0.3312	-0.2080	0.3434	0.2086	-0.0096	-0.1523	0.3800	0.1895	0.3259	-0.2610
L-Leucine	-0.4210	-0.0721	0.2499	0.0851	0.1123	-0.1816	0.4945	0.3031	0.3180	-0.2678
L-Isoleucine	-0.4467	-0.1075	0.2881	0.0897	0.1705	-0.1945	0.5086	0.3223	0.3027	-0.2875
L-Tyrosine	-0.1529	-0.0356	0.0854	0.1036	0.0432	-0.3796	0.5217	0.4968	0.3219	-0.2634
L-Phenylalanine	-0.3493	-0.0490	0.1602	0.0273	0.0778	-0.2685	0.5219	0.4158	0.2951	-0.2251
L-Tryptophan	-0.2633	0.0441	0.2253	-0.0515	0.5000	0.2681	-0.0351	-0.2204	-0.0788	-0.1637
L-Threonine	-0.3492	-0.0477	0.1593	0.0262	0.0795	-0.2735	0.5276	0.4215	0.2930	-0.2252
L-Glutamine	-0.3633	-0.0599	0.1714	0.0663	0.0854	-0.2836	0.5499	0.4142	0.2969	-0.2345
L-Aspartic acid	-0.3805	-0.1432	0.2797	0.1663	0.0492	-0.2161	0.4988	0.2784	0.3035	-0.2468
L-Glutamic acid	-0.3183	-0.1310	0.2863	0.2183	-0.0598	-0.1347	0.3756	0.1567	0.3293	-0.2324
L-Ornithine	-0.3354	-0.1677	0.2674	0.2618	0.0550	-0.2077	0.4442	0.2034	0.2157	-0.2187
L-Histidine	-0.3145	0.0101	0.1005	-0.0264	0.0794	-0.2695	0.5450	0.4337	0.2790	-0.2017
L-Lysine	-0.4986	-0.2594	0.1192	-0.0165	0.1460	0.2356	-0.0207	-0.1680	-0.0616	0.1450
L-Arginine	-0.3380	0.0144	0.1627	0.0251	0.1194	-0.2837	0.5573	0.4246	0.2846	-0.2439
L-Methionine	-0.3553	-0.0633	0.2082	0.0516	0.0451	-0.2245	0.5449	0.3524	0.2983	-0.2287
L-Proline	0.2662	-0.5217	0.1725	0.0370	-0.2708	-0.0644	-0.2180	-0.1023	-0.1050	0.2259
Gamma-aminobutyric acid	-0.3760	-0.2323	0.3934	0.2045	-0.0283	-0.0681	0.3370	0.0974	0.3080	-0.2437
Histamine	-0.3724	-0.0721	0.1901	0.1128	0.0846	-0.2904	0.5866	0.3988	0.2847	-0.2406

Tyramine	0.5677	-0.2981	0.1864	0.5214	-0.2865	-0.1465	-0.1911	-0.0885	0.2176	-0.2367
Putrescine	0.3436	-0.1183	0.0256	0.5180	-0.0934	-0.4540	0.2692	0.3317	0.2561	-0.2952
Ion ammonium	-0.4520	0.4648	-0.0254	-0.2197	0.3652	0.4846	-0.0866	-0.3531	-0.1519	-0.0047
Agmatine sulfate salt	-0.4131	-0.0885	0.2487	0.0846	0.0740	-0.2017	0.4937	0.3193	0.3139	-0.2456
